# Key Success Factors for Regenerative Medicine in Acquired Heart Diseases

**DOI:** 10.1007/s12015-020-09961-0

**Published:** 2020-04-15

**Authors:** Philippe Hénon

**Affiliations:** CellProthera SAS and Institut de Recherche en Hématologie et Transplantation, CellProthera SAS 12 rue du Parc, 68100 Mulhouse, France

**Keywords:** Cell therapy, Regenerative medicine, Myocardial infarction, Acquired heart diseases, Key success factors

## Abstract

Stem cell therapy offers a breakthrough opportunity for the improvement of ischemic heart diseases. Numerous clinical trials and meta-analyses appear to confirm its positive but variable effects on heart function. Whereas these trials widely differed in design, cell type, source, and doses reinjected, cell injection route and timing, and type of cardiac disease, crucial key factors that may favour the success of cell therapy emerge from the review of their data. Various types of cell have been delivered. Injection of myoblasts does not improve heart function and is often responsible for severe ventricular arrythmia occurrence. Using bone marrow mononuclear cells is a misconception, as they are not stem cells but mainly a mix of various cells of hematopoietic lineages and stromal cells, only containing very low numbers of cells that have stem cell-like features; this likely explain the neutral results or at best the modest improvement in heart function reported after their injection. The true existence of cardiac stem cells now appears to be highly discredited, at least in adults. Mesenchymal stem cells do not repair the damaged myocardial tissue but attenuate post-infarction remodelling and contribute to revascularization of the hibernated zone surrounding the scar. CD34^+^ stem cells - likely issued from pluripotent very small embryonic-like (VSEL) stem cells - emerge as the most convincing cell type, inducing structural and functional repair of the ischemic myocardial area, providing they can be delivered in large amounts via intra-myocardial rather than intra-coronary injection, and preferentially after myocardial infarct rather than chronic heart failure.

## Introduction

The new concept of “regenerative medicine” has been up to now mostly applied to cardiac diseases. The current consensus suggests that autologous adult/progenitor cells should be used, at least in the immediate future [1]. The work of Orlic et al. has documented the efficacy of murine lineage-negative c-kit-positive (Linˉ c-kit^+^) bone marrow (BM) cell transplantation for repairing experimentally induced myocardial infarct, resulting in clinical applications for patients with acute myocardial infarct (AMI) [2]. Various sources of stem cells have been proposed. Though initially designed to test the feasibility and safety of cell therapy procedures in several tens of patients, phase I studies using BM-derived cells have shown promise in terms of improved cardiac function and clinical outcomes. From these very preliminary studies, more than 3000 patients suffering from myocardial infarct (MI), ischemic or non- ischemic heart failure (IHF/NIHF), or refractory angina (RA) have now been treated by cell therapy, mostly in randomized controlled trials.

Although these trials have varied widely in design, they have confirmed the feasibility and safety of cell reinfusion procedures. However, the improvement of heart function was globally much less than expected. These divergent outcomes can be explained by differences in the types, sources, and doses of reinjected stem/progenitor cells, cell injection route and timing, choice of efficiency criteria, previous or concomitant reperfusion of the infarcted zone, and type of cardiac disease (AMI, IHF, NIHF, RA), that, separately or together, may have influenced the results of these studies. The aim of this review is to identify key factors which could improve the efficacy of cell therapy in cardiac diseases.

### Types/Sources/Amounts of Cells

#### Myoblasts

Post-MI in-scar transplantation of in vitro-expanded skeletal myoblasts was first proposed in humans based on promising experimental animal data [3]. However, the results of clinical trials did not reliably reproduce a similar improvement in left ventricular (LV) function in humans. Patients with severe post-ischemic LV dysfunction were recruited in several miscellaneous phase-I studies after coronary by-pass graft (CABG) surgery and received trans-epicardial myoblast injections [4–7]. Six months after the procedure, most patients showed moderate improvements in left ventricular ejection fraction (LVEF) and regional wall motion. However, episodes of sustained ventricular arrhythmia (VA) occurred in 15 to 20% of patients. Moreover, LV dysfunction again increased over several months, leading to doubts about the true capacity of myoblasts to reduce ventricular dilatation [5].

In the first randomized, placebo-controlled MAGIC study, patients with IHF received intra-myocardial (IM) injection of 400 or 800 × 10^6^ expanded skeletal myoblasts or placebo at the end of a CABG operation [8]. Six-month data analyses showed a significant reduction in LV volumes in cell-treated patients relative to placebo, but no difference in LVEF. This trial was also hindered by the frequent occurrence of severe VA after cell injection, even though a cardioverter-defibrillator had been previously implanted in all patients, leading to a decision by the steering committee to prematurely discontinue the trial.

Myoblasts are lineage-restricted progenitor cells that can only differentiate into skeletal muscle cells but not cardiomyocytes. The observed VA was likely related to differences in electrical conduction between the injected myoblasts and residual viable cardiomyocytes [8].

Overall, such divergent outcomes and increased risk of severe VA have led most investigators to discontinue using myoblasts for cardiac cell therapy.

#### Bone Marrow Mononuclear Cells

BM is a composite tissue that contains a mix of hematopoietic cells at various stages of maturation and a small number of hematopoietic stem cells (HSCs) capable of self-renewal, and a “stroma” represented by fibroblasts, fat, endothelial, and smooth muscle cells, all derived from mesenchymal stem cells (MSCs), also extremely rare.

Almost 60 mostly randomized clinical trials using BM-MNCs have been reported up to now. Most have been compiled in several meta-analyses that have included many of the same trials, while achieving divergent outcomes. Depending on the trial, 50 to 400 ml of BM were harvested by iliac crest puncture and further enriched for MNCs by density-gradient centrifugation, yielding cell doses varying from 4 × 10^6^ up to 600 × 10^6^ MNCs [9, 10]. This last variable is particularly important, as the potentially positive impact of cell therapy may be dose-dependent [11]. In a meta-analysis compiling 13 studies, the mean change in LVEF significantly favoured administering BM-MNCs using cell doses higher than 10^8^ but did not favour BM-MNC therapy using lower doses [9].

Two other meta-analyses concluded that transplantation of BM-derived cells modestly, but significantly, improved LV function, infarct size, and remodelling in patients with IHF relative to standard therapy. One totalled 2625 patients from 50 studies (35 randomized trials, 15 cohort studies) and additionally showed that these benefits persisted over time and were associated with a reduction in the incidence of death and recurrent MI [12]. In the other (1641 patients from 16 randomized trials), younger patients and/or those with a more severely depressed LVEF achieved the largest benefit [13]. However, some trials included in these meta-analyses resulted in neutral findings when considered individually [14–17].

#### CD34^+^ Stem Cells

CD34 is a transmembrane phosphoglycoprotein antigen first identified in 1984 [18]. It has long been considered to be specifically borne by HSCs [18, 19], but is now also well established as a marker of various non-hematopoietic cell progenitor cells [20–23] present in the BM in similar proportions (0.5 to 1% of total CD34^+^ cells for each). Total CD34^+^ stem cells themselves represent approximately 0.5–1% of total BM-MNCs. All these cells are mobilized into the peripheral blood (PB) by the administration of hematopoietic growth factors (HGF). More particularly, both CD34^+^ endothelial and cardiomyocyte progenitor cells, capable of neo-angiogenesis / neovascularization and cardiac muscle regeneration, respectively, can be easily collected in humans from the BM or the PB [24, 25], except in certain cases from diabetic patients [26].

Experimental and physiological data support the use of CD34^+^ stem cells for cardiac repair. At the end of the 90s’, Asahara et al. reported the intra-cardiac homing of CD34^+^ endothelial progenitor cells after experimental AMI in nude mice [27]. Human (hu)CD34^+^ cells delivered to athymic rats undergoing experimental AMI differentiated more abundantly into cardiomyocytes and endothelial cells in the infarcted myocardium than unselected MNCs and augmented ischemic neo-vascularization on day 28 post-transplant. They also exhibited superior efficacy in inhibiting LV remodelling and preserving myocardial integrity and function [20, 28, 29]. A large proportion of CD34^+^ cells isolated from human PB after granulocyte-colony stimulating factor (G-CSF) mobilization stained positively for c-troponin-T when transplanted directly into the scar of rats with experimental AMI, indicating that they may also differentiate into cardiomyocytes [30]. Hu-CD34^+^ cells persisted in the injured heart for up to one year after reinjection in an experimental nude mouse model and contributed to functional recovery [31]. Post-AMI endogenous circulation and intracardiac homing of endothelial and cardiomyocytic progenitor cells may be enhanced by G-CSF administration and prevent LV remodelling and dysfunction [32].

In humans, the release of endogenous CD34^+^ cells by the BM into the blood increases within the first hours following AMI and continues for several days [33]. A return to normal circulating levels occurs within one week, presumably related to the homing and consumption of these cells within the ischemic area [34], confirming the experimental observation of Asahara et al. [27]. Although such release of CD34^+^ cells appears to be a physiological response to AMI, its effect on limiting ischemic scar formation is not actually measurable, and it is clearly unable to compensate for the loss of infarcted cardiomyocytes.

Despite such favourable outcomes, only a few clinical trials have tested selected CD34^+^ cells in AMI. From the end of 2002, our group conducted the first pilot study to use G-CSF-mobilized and immuno-selected PB-CD34^+^ cells in MI patients with a poor prognosis [25]. They received trans-epicardial CD34^+^ cell injections (average: 52 × 10^6^; range: 27–104 × 10^6^) directly into the ischemic scar at the end of a CABG operation for compassionate reasons. All but one patient showed a marked and sustained improvement of LVEF from baseline values four years after the procedure (mean: +21 percentage points, range: +4 to +42), associated with cardiac tissue regeneration, demonstrated by PET, and the recovery of contractility in the previously akinetic area. All these are still alive and well, with a present average follow-up (FU) of 14 years, including three who were initially scheduled for early heart transplantation and have thus far avoided it. Additionally, we detected the presence of small CD34^+^ subpopulations by flow cytometry (FCM) that co-expressed c-TroponinT (a cardiac marker) or CD133^+^/VGEFR-2 (an endothelial marker) in mobilized CD34^+^ cells from the AMI patients, whereas they were almost undetectable in those of controls.

Among randomized studies using CD34^+^ cells in AMI, CD34^+^ cells have been purified and enriched by immuno-magnetic selection either from BM aspirates or PB after mobilization by G-CSF and collection by leukapheresis (LKP) [9, 10].

Once again, the therapeutic results were dose-dependent. Low (1 × 10^3^), mid (1 × 10^5^), and high (5 × 10^5^) doses of hu-CD34^+^ cells were delivered IM in athymic rats after ligation of the left anterior descending (LAD) coronary artery. [30]. Four weeks after transplant, the rats receiving the highest dose of hu-CD34^+^ cells showed a higher potential for vasculogenesis and cardiomyogenesis, with functional recovery from the MI. In humans, intracoronary (IC) injection of a threshold dose >10 million CD34^+^ cells was associated with a significantly greater improvement in LVEF, perfusion, and infarct size than lower doses [24, 35]. The Regent trial compared IC infusion of either BM-CD34^+^/CXCR4^+^ cells or unselected BM-MNCs in patients with severely reduced LVEF: the delivery of small doses of CD34^+^/CXCR4^+^ cells or 100 times more unselected BM-MNCs (1.90 × 10^6^ vs 1.78 × 10^8^) was associated with a similar trend towards an improvement in LVEF [36]. In the randomized phase II PreSERVE AMI study, 8 to 40 × 10^6^ (mean: 14.9 ± 8) autologous BM-CD34^+^/CXCR4^+^ cells or placebo were delivered IC to patients with LV dysfunction post-STEMI [37]. There was a significant dose-dependent reduction in the occurrence of severe adverse events (SAE) and major adverse cardiovascular events (MACE) one year post-injection relative to placebo, but no significant improvement in LVEF, except for patients who received more than 20 × 10^6^ CD34^+^/CXCR4^+^ cells. In our pilot study, a significant average improvement of LVEF of +18 percentage points from baseline values was observed two years after injection of an average of 52 × 10^6^ CD34^+^ cells [25].

However, the effect of high doses of CD34^+^ cells might be less constant in chronic heart failure (CHF) patients. IC injection of an average of 113 × 10^6^ CD34^+^ cells in patients with dilated NIHF was followed by a progressive increase in LVEF values of up to 8% three years after injection, whereas LVEF did not improve in controls [38]. However, the improvement in cardiac function was less with the higher dose (5 × 10^5^/ kg. body weight – b.w.) than the lower dose (1 × 10^5^ /kg. b.w.) in another study comparing the effect of two different CD34^+^ cell doses in RA patients [39]. The low dose resulted in a clinically meaningful durable improvement in total exercise time (TET), frequency of angina, and decreased incidence of mortality and MACE at 24 months, whereas no change in LV function was recorded after endo-cardiac delivery of similar CD34^+^ cell doses in the RENEW trial, unfortunately terminated by the sponsor before its completion due to strategic considerations [40].

#### Mesenchymal Stem Cells (MSCs)

MSCs are still more rare in the BM (1/10,000 MNCs) than CD34^+^ cells. They do not bear the CD34 antigen on their surface, except at their very earliest stage. They can be easily isolated from the BM and enriched and expanded in specific media for 3 to 4 weeks to reach up to 10^8^ cells [41]. MSCs can also be harvested from the umbilical cord [42], Wharton’s jelly [43], and adipose tissue [44]. They have immunomodulatory and immunosuppressive properties (reviewed in [45]). The mechanism of action for their cardioreparative effects is likely multi-factorial: although MSCs do not appear to actually be capable of differentiating into contracting cardiomyocytes in vivo [46], they may 1) enhance myocyte cell cycling [47], 2) inhibit formation of fibrosis in the border zone of the scar [48] and reduce stiffness of the scar [46], thus limiting its secondary extension [49], and 3) secrete either exosomes or potent soluble proangiogenic factors that allow revascularization and reperfusion of the hibernated area surrounding the scar. These mechanisms could thus attenuate post-infarction remodelling [50].

The hypo-immunogenic status of MSCs would make allogeneic transplants feasible and thus permit “off the shelf” use [45]. Among 12 randomized clinical trials reviewed in Jeong et al. [51], five used allogeneic MSCs, either in AMI or CHF. In a phase I randomized study, Hare et al. intravenously reinfused 53 AMI patients with allogeneic BM-MSCs or placebo at a 2:1 ratio [52]. The MSC group showed an improvement in overall clinical status six months after infusion, with fewer arrhythmic events and modestly improved LVEF. In the randomized phase I/II POSEIDON study, which compared allogeneic versus autologous MSC therapy in 30 patients (1/1 ratio) with AMI, the same group further reported that both were safe and showed trends towards reducing infarct size and improving ventricular remodelling [53]. Only a very few mild allo-immune reactions occurred in the two studies. An additional study randomly assigned 20 AMI patients (1:1) after percutaneous coronary intervention (PCI) to receive intravenous (IV) BM-derived allogeneic MSCs (Stempeucel®) or placebo. No adverse toxicity was observed during or immediately after Stempeucel® delivery. However, there was no overall effect of Stempeucel® in improving cardiac function at six months or two years versus placebo [54].

In another study, 45 patients with severe CHF (LVEF ≤40%) were randomly distributed into three equal groups to receive trans-endocardial injection of 25, 75, or 150 × 10^6^ allogeneic mesenchymal progenitor cells (MPCs) versus 15 control patients [55]. At the 12-month FU, only the 150 × 10^6^ group showed a trend towards a small, but non-significant, improvement in TET and a lower incidence of MACE versus placebo. However, there are concerns about the fact that this last end-point was generated in a *post-hoc* manner. Thirteen percent of all MPC patients (and nearly 20% in the 150 × 10^6^ group) developed anti-donor antibodies, but without immediate clinical consequences. In the TRIDENT study, 30 patients with IHF received either 20 or 100 × 10^6^ allogeneic MSCs via trans-endocardial injection in a blinded manner. Although both doses reduced scar size, only the higher dose weakly increased LVEF [56].

Chen et al. reported the first study using autologous BM-MSCs after PCI in AMI patients who were randomized to receive IC injection of 8 to 10 × 10^9^ BM-MSCs or saline. The cell-treated group showed a significant improvement in wall movement velocity over the infarcted region, LVEF, and perfusion defects relative to controls [57]. In two studies with a similar design, STEMI patients were randomly allocated to receive either IC administration of autologous BM-MSCs or standard of care (SOC). Although a modest improvement in LVEF was recorded at the six-month FU in one group, changes in the left ventricular-end diastolic volume (LVEDV) and left ventricular-end systolic volume (LVESV) did not significantly differ between groups [58]. In the second study, no significant differences in myocardial viability or myocardial perfusion within the infarct area or LVEF were observed [59].

In the MSC-HF trial, patients with severe IHF were randomized 2:1 for IM injections of autologous BM-MSCs or placebo (PBS). At the six-month FU, the LVESV was significantly lower in the MSC group and higher in the placebo group. There were also a significant improvement in LVEF, stroke volume, and myocardial mass measured by MRI relative to the placebo group. [60]

#### Cardiac Stem Cells (CSCs)

The heart has long been considered to be a post-mitotic organ, incapable of self-regeneration. However, several investigators have made the hypothesis that the heart contains various amounts of undifferentiated cells (characterized by their being *c-kit* positive), and postulated that these cells may be cardiac stem cells (CSCs), the activation of which would lead to the formation of new myocardium [61]. This concept arose from the initial observations of Orlic [2] that have generated subsequent criticism, calling it into question [62, 63]. Nonetheless, the field amazingly shifted its focus towards endogenous c-kit^+^ CSCs that reside within the myocardium [64].

In the SCIPIO Phase I trial, autologous c-kit^+^ “CSCs”, previously isolated from endomyocardial biopsies, expanded for 41 days, and immunomagnetically sorted, were IC re-injected versus placebo after CABG to patients with ischemic cardiomyopathy [65]. Initial results showed a small, albeit significant, improvement in LVEF and infarct size in “CSC”-treated patients only. However, there is doubt concerning the actual nature of what the authors called “CSCs”, as their immuno-phenotype (Lin^−^ c-kit^+^, with endothelial and myocytic subpopulations) is close to that of CD34^+^ cells [66]. Within hours/days after the occurrence of AMI, CD34^+^ cells are spontaneously mobilized from the BM into the peripheral blood and migrate to the myocardium, where they have the capacity to colonize for a certain time [33, 34]. Thus, “endogenous CSCs” might actually be CD34^+^ cells scattered throughout the myocardial tissue and still able to expand or differentiate into endothelial and cardiomyocytic progenitor cells [25]. This hypothesis is supported by the results of two recent experimental studies that concluded that adult hearts contain no or extremely few CSCs [67, 68]. Moreover, serious concerns about the integrity of data contained in the SCIPIO study have led to an “Expression of Concern” issued by the editors of *The Lancet,* and 31 articles from the same group, assessing the existence of CSCs, have been recently retracted due to charges of fraud.

In the CADUCEUS trial, autologous cells harvested from endomyocardial biopsies performed percutaneously in patients with moderate and generally presymptomatic LV dysfunction were grown in suspension cultures to enable the self-assembly of three-dimensional “cardiospheres” [69]. Their subsequent re-plating on adherent culture flasks yielded cardiosphere-derived cells (CDCs), which were finally re-infused into the infarct-related artery. MRI analysis of patients treated with CDCs showed a reduction in scar mass, increase in viable heart mass and regional contractility, and wall thickening at six months relative to controls, but there was no change in LVEF, LVEDV, or LVESV, which were the primary endpoints. The mechanism of the potential impact of CDCs on cardiac function was postulated to be indirect, with paracrine stimulation of endogenous cells, which would correlate well with the fact that “CDCs” are mainly composed of MSCs, associated with a few endothelial and hematopoietic progenitor cells. However, the ALLSTAR trial subsequently launched by the same group, in which patients with prior MI were randomized to receive IC injection of proprietary allogeneic CDCs (CAP-1002) versus placebo, failed to show in an interim analysis a reduction of infarct size (chosen as the primary objective based on results of the CADUCEUS trial) or an improvement in LV volumes [70]. The trial was halted by decision of the sponsor due to the low probability that a treatment effect would be observed and ongoing follow-up was officially ceased on February 28, 2019 (Clinical trials.gov),

#### “Other” Cells

An original approach has been developed in the C-CURE randomized Phase II study [71]. Cardiac progenitor cells (‘“cardiopoietic cells”) were differentiated from autologous BM-MSCs, ex-vivo expanded, and exposed to a proprietary cardiogenic cocktail. Patients with IHF received SOC or SOC plus cardiopoietic cells (733 × 10^6^ on average) delivered by 9 to 26 endomyocardial injections with the NOGA system into the hibernated myocardium surrounding the scar. At the six-month FU, the cell-treated group showed an improvement in LVEF and TET and a reduction of LVESV versus the SOC group, but without regeneration of the fibrotic scar. However, these results have now been called into question, as numerous discrepancies in the paper have been noted since its publication and in reports of this study in various scientific meetings [72].

Furthermore, final results of the subsequent Phase III CHART-1 clinical trial, conducted by the same group with the aim to validate the efficacy and safety of cardiopoietic cells delivered via a retention-enhanced injection catheter in advanced IHF, failed to reach its primary efficacy endpoint at 39 weeks FU, leading the sponsor to definitively abandon the cardiac field [73]. However, 15 of the 58 initial CHART-1 investigators amazingly published six months after a *post-hoc* analysis showing that IM administration of cardiopoietic cells led to a progressive decrease in LVEDV and LVESV through 52 weeks of FU [74].

The main clinical trials according to cell types are listed in Table 1.

**Table 1:** List of cell therapy clinical trials in cardiac diseases according to the type of cells used

Cell types	Studies	Auto Allo	Random	Diseases	Average Cell doses	Route	Number of patients	Results	F.U.
**Myoblasts**									
	MAGIC *Menasché* et al. [8]	Auto	Yes:3 groups C; LD; HD	MI	LD: 4 .10^8^ HD: 8.10^8^	IM	Subjects: 97 Controls: 30	Neutral	6 months
**BM-MNCs**									
	Meta-Analysis *Martin-Rendon* et al. [9]	Auto	Yes: 2 groups C; MNCs	AMI	1.10^7^ to 2,5.10^9^	IC	Subjects: 326 Controls: 312	LVEF: ↗ LVESV: ↗	6 to 12 months
	Meta-Analysis *Brunskill* et al. [10]	Auto	Yes: 2 groups C; MNCs	AMI/CHF	10^7^ to 10^8^	IC:20 trials IM: 5 trials	Subjects: 565 Controls: 526	IC - LVEF: 0 IM - LVEF: ↗ AMI > IHD	6 months
	Am Heart J 2006 *Meluzin* et al. [11]	Auto	Yes: 3 groups C; LD; HD	AMI	LD: 10^7^ HD: 10^8^	IC	66 (22 in each group)	LVEF ↗ in HD LVESV↘ in HD	3 months
	Meta-Analysis *Jeevanantham* et al. [12]	Auto	Yes: 35 trials No: 15 trials	AMI/CHF	10^6^ to 10^9^	Trials Nb: IC:38 IM:12	Subjects:1460 Controls1165	LV function ↗ Remodelling ↗	4 to 36 months
	Meta-Analysis *Delewi* et al. [13]	Auto	Yes:2 groups C; MNCs	AMI	50–400.10^6^	IC	Subjects: 984 Controls: 657	LV function ↗ in younger and severe patients	3 to 6 months
	ACCRUE Meta-Analysis *Gyöngyösi* et al. [89]	Auto	Yes (Prospective) C; MNCs	AMI	150 .10^6^	IC	Subjects: 767 Controls: 485	Neutral	3 to 12 months
	REPAIR-AMI trial *Schächinger* et al. [93]	Auto	Yes: 2 groups C; MNCs	AMI	198.10^6^	IC	Subjects: 101 Controls: 103	LVEF↗ only in more severe patients	12 months
	BOOST trial *Meyer* et al. [95]	Auto	Yes: 2 groups C; MNCs	AMI	24,6 ± 9,4.10^6^	IC	Subjects: 30 Controls: 30	Neutral	18 months
**34**^**+**^**SCs**									
	POC Study *Pasquet* et al. [25]	Auto (PB)	No	AMI	52.10^6^	IM	Subjects: 7	LVEF ↗↗↗ Cardiac repair	24 months
	Amer Heart J 2011 *Quyyumi* et al. [24]	Auto (BM)	Yes: 4groups LD; MD; HD C	AMI	5;10;15.10^6^	IC	Subjects: 16 Controls: 15	LVEF ↗↗ if 34^**+**^ SCs ≥ 10^7^	6 months
	REGENT *Tendera* et al. [36]	Auto (BM)	Yes: 2 groups MNC, 34^**+**^**SC**	AMI	MNC:1,8.10^8^ 34^**+**^:1,9.10^6^	IC	MNCs: 80 34^**+**^ SCs: 80	Neutral	6 months
	PRESERVE – AMI *Quyyumi* et al. [37]	Auto (BM)	Yes C; 34^**+**^SC	AMI	8–44.10^6^	IC	Subjects: 78 Controls: 83	LVEF ↗↗ if 34^+^SCs ≥20 × 10^6^	12 months
	Circ Res 2013 *Vrtovec* et al. [38]	Auto (PB)	Yes C; 34^+^SC	CHF	113 ± 26.10^6^	IC	Subjects: 55 Controls: 55	LV function↗ TET ↗	60 months
	Circ Res 2011 *Losordo* et al. [39]	Auto (PB)	Yes:3 groups C; LD; HD	RA	LD: 1.10^5^/kg HD:5.10^5^/kg	IM	Subjects: 111 LD: 55 HD: 56 Controls: 56	Less angina crisis (LD) TET ↗ (LD)	12 months
	RENEW trial *Povsic* et al. [40]	Auto (PB)	Yes:3 groups SOC; C; 34^**+**^SC	RA	1.10^5^/kg	IM	Subjects: 50 SOC: 6 Controls: 28	Less angina TET ↗ Less MACE	Early closure
**MSCs**									
	Meta-analysis *Jeong* et al. [51]	Auto [9] Allo [5]	Yes: 2 groups C, MSCs	AMI: 6 IHF: 6	Auto:1–25.10^6^ Allo: 2–72.10^6^	IM:4 trials IC: 5 trials IV: 3 trials	Subjects: 509 Controls: 441	LVEF ↗ Mass scar: **-** 1.13	24 months
	JACC 2009 *Hare* et al. [52]	Allo (BM)	Yes: 4 groups C; LD; MD; HD	AMI	LD: 0,5.10^6^/kg MD:1,6,10^6^/kg HD: 5.10^6^/kg	IV	Subjects: 39 Controls: 21	LVEF ↗	6 months
	POSEIDON trial *Hare* et al. [53]	Auto vs Allo (BM)	Yes dose ranging: 6 sub-groups	IHF	LD: 20.10^6^ MD: 100.10^6^ HD: 200.10^6^	IM	Auto: 15 Allo: 15	Trends towards infarct size reduction and reverse remodeling	12 months
	Cytotherapy 2015 *Chullikana* et al [54]	Allo (BM)	Yes C, MSCs	AMI	2.10^6^/kg	IV	Subjects: 10 Controls: 10	Neutral	24 months
	Circ Res 2015 *Perin* et al. [55]	Allo (BM)	Yes: 4 groups LD; MD;HD C	IHF NIHF	LD: 25.10^6^ MD: 75.10^6^ HD: 150.10^6^	IM	Subjects: 45 Controls: 15	LVESV LVEDV ↘ with HD	36 months
	TRIDENT Study *Florea* et al. [56]	Allo (BM)	Yes: 2 groups LD, HD	MI	LD: 20.10^6^ HD: 100.10^6^	IM	LD: 15 HD: 15	Scar size reduction LVEF ↗ in HD	12 months
	Am J Cardiol 2004 *Chen* et al. [57]	Auto (BM)	Yes: 2 groups C, MSCs	AMI	8–10.10^9^	IC	Subjects: 35 Contols: 34	Wall movement ↗ LVEF ↗	24 months
	Inter J Cardiol 2013 G*ao* et al. [59]	Auto (BM)	Yes: 2 groups C, MSCs	AMI	3,08 ± 0,52.10^6^	IC	Subjects: 21 Controls: 22	Neutral	12 months
	MSC--HF trial *Mathiasen* et al. [60]	Auto (BM)	Yes: 2 groups C, MSCs	MI	77.5 ± 67.9.10^6^	IM	Subjects: 40 Controls: 20	LVESV ↘ LVEF ↗	6 months
**CDCs**									
	CADUCEUS trial *Makkar* et al. [69]	Auto (Cardiac Biopsy)	Yes: 2 groups C; CDCs	MI	12,5–25.10^6^	IC	Subjects: 17 SOC: 8	Scar mass ↘ Wall thickening	6 months
**CPCs**									
	C - CURE trial *Bartunek* et al. [71]	Auto (BM)	Yes: 2 groups SOC, CPCs	ICHF	≥ 600 .10^6^	IM	Subjects: 32 Controls: 15	LVEF ↗ TET ↗ LVESV ↘	6 months
	CHART-1 trial	Auto (BM)	Yes: 2 groups Sham, CPCs	ICHF	≥ 600 .10^6^	IM			
	*1-Bartunek* et al. [73] *2- Teerlink* et al. [74]						Subjects: 120 Sham: 151 Subjects: 157 Sham: 158	Neutral primary endpoint LVESV↘ LVEDV↘	39 weeks 52 weeks

**Abbreviations**: Auto/Allo, autologous/allogeneic; F.U., follow-up; BM, bone marrow; PB, peripheral blood; BM-MNCs, bone marrow mononuclear cells; 34^+^ SC, CD34^+^ stem cells; MSCs, mesenchymal stem cells; CDCs, cardiosphere-derived cells; CPCs, cardiopoietic cells; C, controls; LD/ MD/HD, low/mid/high doses; MI, myocardial infarct; AMI, acute myocardial infarct; CHF, chronic heart failure; RA, refractory angina; IHF, ischemic heart failure; NIHF, non-ischemic heart failure; IM/IC/ IV, intra-myocardial/intra-coronary, intra-venous cell delivery; LV, left ventricle; LVEF, left ventricle ejection fraction; LVESV, left ventricle end systolic volume; LVEDV, left ventricle end diastolic volume; TET, total exercise time; MACE, major adverse cardiovascular events; SOC, standard of care; POC, proof of oconcept. ↗/↗↗/↗↗↗, weak/ significant/ highly significant increase. ↘, weak decrease

### Route of Delivery

Two routes of cell delivery have been mainly used: IC or IM, whereas the IV route was only used in a few MSC trials.

The IC route offers the advantage of being a non-surgical method compared to the direct IM delivery during CABG performed in preliminary studies. It can be performed percutaneously during angioplasty or stenting. However, percutaneous endomyocardial stem cell delivery through percutaneous injection catheters with a needle incorporated at the top may become the method of choice. Cell reinjection is then performed either after completion of ventriculograms obtained by bi-plane angiography (Helix™catheter, Biocardia Inc.,South San Francisco, CA) [75, 76] or three dimensional LV electromechanical mapping to identify the foci of the ischemic myocardium (Myostar™ Catheter combined with the guidance system NOGA®, Biologics Delivery Systems, Diamond Bar, CA) [77, 78]. Such technology appears to be feasible and safe, avoids surgery, and would make IM delivery as easy as IC delivery [79]. Furthermore, unexpected high rates of coronary in-stent restenosis after primary angioplasty and IC delivery of G-CSF-mobilized blood stem cells suggests caution in using the IC route for this indication [80].

Data from three studies using various radioactive cell markers have shown limited survival of cells in the myocardium after IC injection. Only 1.3 to 2.6% of unselected ^18^F-FDG labelled BM-MNCs was actually retained in the infarct center and border zone one hour after IC transfer, whereas CD34^+^-enriched cells displayed a higher retention rate (14 to 39%) but predominantly homed to the border zone of the scar [81]. Two other studies confirmed both significant, but more limited, uptake in the border zone of CD34^+^-enriched cells after IC transfer, varying from 5.5 [82] to 9.2% [83] of the total radioactivity after one hour. Such cell localisation would favour the neo-revascularization process of the border zone but would not be sufficient to trigger regeneration of the damaged myocardial tissue. In all three studies, the remaining radioactivity was distributed mainly between the liver, spleen, and bone marrow, regardless of cell type. Furthermore, myocardial radioactivity decreased over time, although 6.8% of the total radioactivity was still expressed 24 h after IC delivery [83].

Cell survival after IM injection has only been evaluated in experimental studies. In an ischemic mouse model, significantly more BM-MNCs were retained in the heart after IM injection than after IC injection (11.3 ± 3% vs 2.6 ± 0.3%) [84], but there was also greater variability in the locally delivered doses. A consistently higher retention rate (57%) was observed one hour after injection of neonatal rat cardiomyocytes transferred into adult rat ischemic myocardium [85]. Although 48% of skeletal muscle precursor cells still survived 10 min after implantation, only 14.6% were still alive by 24 h [86]. Mechanical leakage and washout may account for most cell loss after IM implantation, which may be higher in beating than non-beating hearts [87].

Clinical data appear to confirm that cells injected directly into the damaged myocardium or the border zone are retained more efficiently than cells infused via coronary arteries. The results of several clinical trials or meta-analyses have shown that IC infusion of BM-MNCs, as well as MSCs, did not improve LV function [15, 16, 88]. More particularly, the ACCRUE meta-analysis, the only prospectively declared collaborative multinational database that includes individual data of patients with AMI from 12 randomized trials, showed that IC cell therapy provided no benefit in terms of clinical events or changes in LV function and remodelling [89]. On the contrary, trans-endocardial CD34^+^ cell transplantation into dysfunctional myocardium is associated with higher myocardial retention rates and greater improvements in ventricular function, LVEF, heart remodelling, and exercise capacity in patients with AMI or dilated IHF than by the IC route [10, 90, 91].

The IV route has been the most often used in experimental small-animal studies, with conclusive results. However, a pig model to compare IC versus IV administration of ^99^mTC–BMMNC showed cardiac radioactivity to be almost null (0.16 ± 0.23%) one hour after IV cell delivery compared to that of pigs after IC cell delivery (34.8% ± 9.9%), and completely disappeared after 24 h, suggesting the absence of cardiac homing when using the IV route [92]. In humans, Hare et al. [52] and Chullikana et al. [54] conducted randomized double blind, placebo-controlled, dose-escalation or single-dose studies respectively, of IV adult human MSC injection after AMI. As the IV route was exclusively used in these studies, it is impossible to determine whether it was at least partially responsible for the weak or absent improvement of cardiac function reported in both.

### Timing of Cell Injection

The timing of cell injection relative to the occurrence of AMI is also an important factor. The meta-analysis performed by Martin-Rendon et al. that have reviewed randomized clinical trials in which autologous BM-MNCs were delivered IC, suggested that the improvement in LVEF was significantly greater if the treatment was administered later than seven days after the AMI than if it was administered within the first week [9]. This was further confirmed by two other individual clinical studies [93, 94]. This may be explained by the fact that the infarct-related inflammatory process is strongest in the first days after the onset of AMI and would offset the putative benefits of the early administration of stem cells due to the loss of their therapeutic quality. In contrast, if the treatment is administered too long after AMI, scar formation associated with fibrosis will progressively reduce the benefit of cell therapy. This probably explains the very moderate improvements - if any - reported when cell therapy was administered in patients with IHF or refractory angina post-AMI [30]. However, the development of scar fibrosis is relatively slow and takes several months to be fully achieved.

The ideal time for the application of cell therapy is thus likely between the eighth day and the end of the second month after AMI, even if successful application has been observed for up to six months after [25].

### Severity/Stage of Heart Disease

#### AMI

For safety reasons, patients in most clinical trials had relatively well-preserved ventricular function with a low risk of death or development of secondary CHF, creating a challenge for demonstrating significant improvements in cardiac function. However, several studies have assessed the importance of baseline LVEF on cell therapy.

The REPAIR-AMI study showed that BM-MNC-treated patients with a baseline LVEF ≤48.9% had significantly greater improvement in LVEF than those above this value [93]. A similar trend was observed in the REGENT study in patients with a median baseline LVEF <37% [36]. In the BOOST study, patients with a larger transmural AMI achieved greater improvement of LVEF than those less severely affected at six months FU, although this was not sustained at 18 months [95]. Delewi et al. also concluded that patients with a more severely depressed LVEF at baseline derived more benefit from IC BM-MNC therapy [13].

Although somewhat less significant, the meta-analysis performed by Brunskill et al., subdivided 15 trials into two groups, either with a median baseline LVEF below 48.5%, (Group A), or above the median (Group B). A significant improvement in LVEF was observed in both groups, but amazingly, Group-A patients showed a smaller (3.19% vs 8.67%) although more significant effect (*p* = 0.0005 vs *p* = 0.03) than group-B patients likely due to the larger number of studies (nine vs six) and participants (499 vs 297) for Group A than Group B [10]. The enrolment of AMI patients with a poor prognosis and the most favourable risk-benefit ratio is thus favoured [96, 97].

#### Chronic Heart Failure

Around 15 clinical trials assaying cell therapy in CHF have been completed, for a total of approximately 900 patients with NYHA functional class II to IV symptomatic CHF or Canadian Cardiovascular Society class III/IV angina, refractory to medical treatment with no conventional percutaneous or surgical revascularization option. Most studies were randomized and placebo controlled. In three, patients were assigned to receive immuno-selected CD34^+^ cells or placebo [38, 39, 77], whereas in the others, patients received unselected BM-MNCs or placebo [98, 99]. The cells were mostly delivered via direct catheter-based endomyocardial injections using either the NOGA™ System [39, 40, 55, 76, 77, 80, 99–101], or the Biocardia Helix™ catheter [53, 102], or via the IC route [38, 103, 104]. Trans-epicardial BM-MNC implantation was performed as an adjunct to CABG surgery in four studies [105–108].

Globally, BM-MNC patients received 17 to 132 × 10^6^ unselected MNCs, containing an average of 1.81 × 10^6^ CD34^+^ cells, when determined (range: 0.8 to 2.9 × 10^6^). Cell-treated patients showed a moderate, but significant decrease in angina frequency and improvement in myocardial perfusion and exercise tolerance six months after cell reinfusion relative to their baseline data or controls in nine studies. A trend towards a decrease in LVEDV and LVESV was often observed. LVEF significantly improved in six studies [38, 99, 102, 105–107] but not in the others [39, 76, 102]. The injection of MNCs directly into the scar or artery supplying the scar during CABG surgery did not improve contractility of the non-viable scarred myocardium, reduce scar size, or improve LVEF more than CABG alone [105]. In addition, the phase II FOCUS trial enrolled 92 patients with coronary artery disease or left ventricular dysfunction and limiting heart failure or angina, who were randomized at a 2:1 ratio to receive trans-endocardial injection of 100 × 10^6^ autologous BM-MNCs or placebo [109]. There were no significant changes in LVESV, maximal oxygen consumption, or reversible defects at six months and the authors concluded that a shot of BM-MNC therapy is of no use in CHF.

However, several studies with a longer FU have slightly tempered such a negative conclusion. A meta-analysis reporting data from 492 patients compiled from 11 randomized clinical studies concluded that cardiac dysfunction and remodelling improved significantly with IM delivery of BM-MNCs and that the therapy was more efficacious in patients who were candidates for CABG than in those who were not [110]. IHF patients were randomized at a 1:1 ratio to intramyocardially receive autologous BM-MNCs vs controls. At the 12-month FU, 21/55 patients in the control group had died vs 6/54 in the treated group. [101]. BM-MNCs were non-randomly injected IC into 191 CHF patients vs 200 who received SOC. At the 60-month FU, seven patients in the treated group had died versus 32 in the control group [103]. The total mortality at the five-year FU was also lower for patients who received 113 ± 26 × 10^6^ CD34^+^ cells IC than controls (14% vs 35%) [29]. Two IC infusions (four months apart) of high dose autologous BM-MNCs (1533 ± 765 × 10^6^ BM-MNCs, including 23 ± 11 × 10^6^ CD34^+^ cells and 14 ± 7 × 10^6^ CD133^+^ cells) were performed in a non-randomized manner on 32 patients with chronic systolic dysfunction (LVEF 33 ± 9%) [104]. According to the median number of CD34^+^ cells they received, there was a trend towards decreased mortality (3 vs 7 patients), readmission rate (5 vs 7 patients), and morbidity at the seven-year FU for patients who received 32 ± 9 × 10^6^ CD34^+^ cells versus those who received15.5 × 10^6^ CD34^+^ cells.

## Discussion

Stem cell therapy offers a breakthrough opportunity for the improvement of severe heart diseases and many individual clinical trials have been performed since the early 2000’s to assess such improvement, but the results have been conflicting. Most meta-analyses that have compiled individual studies appear to confirm significantly positive but variable effects, depending on the analysis. These meta-analyses must be interpreted with caution, as they were hampered by many discrepancies that certainly affected the results: differences in trial design and sample size, cell type and source, route of injection, cell doses reinjected, disease indication (AMI, IHF, NIHF, RA), and duration of FU [111, 112]. However, the results of such meta-analyses can still aid in the design of future clinical trials [113], and several crucial key factors that favour a successful strategy for the cell therapy of ischemic heart diseases emerge from a review of their data.

### Definition of the Best Cell Type

First, it is time to correct a misconception concerning BM-MNCs: contrary to the long-standing belief of many clinical investigators who misunderstood the results of Orlic’s study [2] and which has now persisted for more than a decade, BM-MNCs are not “stem cells”, but a mix of various cells of hematopoietic lineages at various stages of maturation and stromal cells. They also contain, but only in very low numbers, cells that have stem-cell-like features, i.e. capable of either long-term self-renewal or differentiation (1% CD34^+^ cells and 1/10,000 MSCs). For example, the number of CD34^+^ cells has varied from 0.18 × 10^6^ in 24 × 10^6^ up to 4.2 × 10^6^ in 600 × 10^6^ MNCs (average 1.8 × 10^6^) when they have been analysed in clinical trials using BM-MNC [9, 11]. As a severe AMI destroys between 1 and 2 billion cardiomyocytes, it is clear that reinfusing several hundred thousand or, at the best, a few million potentially active cells cannot efficiently replace such cell loss. This would at least partially explain the neutral results [109], or at best the modest improvements in LV function when more than 10^8^ MNCs were delivered, coming from tens of clinical trials using MNCs, enrolling thousands of patients, and at high cost in terms of finances and investigators time. Furthermore, these disappointing results have contributed to the deep scepticism of many cardiologists concerning the actual potential of cell therapy in AMI and IHF.

The capacity of MSCs to improve LV function after AMI is still a subject of debate. MSC injection in/around the infarct scar could result in a limited improvement in LVEF and ventricular remodelling after AMI or IHF. They likely inhibit the formation of fibrosis and stiffness of the border zone of the scar and allow revascularization of the hibernated zone surrounding it [114]. Their hypo-immunogenic status may have been advantageous, allowing “off the shelf” allogeneic use. However, there is experimental evidence to suggest that cellular and humoral anti-donor responses in recipients can sensitize them to subsequent allo-antigen exposure, which should not be underestimated in a patient population that may require further cardiac transplantation [46]. Allo-sensitization may also limit MSC longevity and attenuate their beneficial effects. Wu et al., who tracked transplanted BM-MSCs, observed that they survived in the heart for less than five weeks after transplantation and did not contribute to tissue regeneration [115]. The only advantage of adipose or Wharton’s jelly MSCs upon BM-MSCs is easy accessibility [116, 117]. In addition, the risk of adverse events specific to MSCs, such as bone-tissue formation in the infarcted heart [118] or occurrence of microvascular thrombosis [119, 120], has been suggested**.**

The true existence of cardiac stem cells now appears to be highly discredited, at least in adults [67, 68], as they are more likely CD34^+^ cells deposited into the myocardium after AMI still capable to differentiate along the cardiac or endothelial pathways [66]. Endomyocardial biopsy sampling is an invasive procedure that cannot be ethically justified to collect these cells of interest, when they can be easily collected in much larger amounts from circulating blood [25]. This also applies to cardiosphere-derived cells, which mainly consist of MSCs and fail to improve heart function after MI [70].

Lineage-specified “cardiopoietic cells” lack self-renewal capacity, which makes their long-term survival uncertain and would consequently contribute to progressive exhaustion of their potential angiogenic or positive paracrine effect, diminishing the persistence of any improvement in heart function. The numerous discrepancies recorded in the report of the C-Cure study [72] and the ambiguity of the two papers successively published on the Chart-1 study, with contrasting conclusions [73, 74], make it difficult to evaluate the actual therapeutic potential of cardiopoietic cells, all the more so as the sponsor has decided to definitively halt their production.

**Fig. 1:**
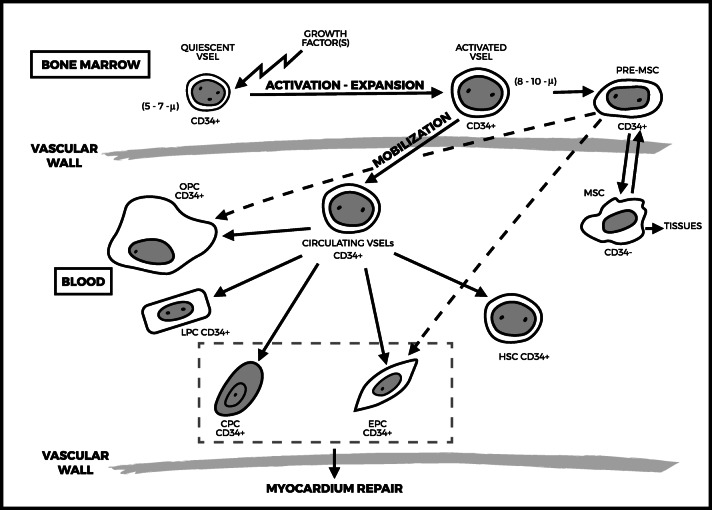
Proposed schema of developmental interrelationship between very small embryonic-like stem cells (VSELs), and tissue-committed cells. Quiescent VSELs deposited longlife into the bone marrow, may migrate into the blood once activated and give rise not only to HSCs and EPCs but also to other tissue-committed cells, and are also a source of mesenchymal stem cells (MSCs). More particularly, their capability to differentiate along both the cardiac and endothelia pathways favours their clinical use in cardiac diseases. Dotted line pathway still under investigation. Abbreviations: MSC, mesenchymal stem cell; HSC, hematopoietic stem cell; EPC, endothelial progenitor cell; CPC, cardiac progenitor cell; LPC, liver progenitor cell; OPC, osteoblastic progenitor cell

Thus CD34^+^ cells emerge as the most convincing cell type among those which have been experimentally and clinically evaluated for their potential ability to compensate for AMI-related cardiomyocyte loss. The transgressive hypothesis that CD34^+^ HSC can transdifferentiate into other lineages, and more particularly functioning cardiomyocytes, has often been suggested over the last decade [20, 21, 121, 122], starting from the initial Orlic’s publication, although it had been further severely refuted by other investigators [62, 63]. However, such concept of cellular “plasticity” may have to be rejected [123], as it now appears more likely that all CD34^+^ cell sub-populations may be derived from very small embryonic-like (VSEL) stem cells deposited during ontogenesis that reside for life in the BM. VSELs were first identified and characterized by their very small size (from 3 to 5 μm in diameter) in murine adult tissues [124], and further in human BM (5 to 6 μm in diameter) [125]. These cells represent a rare (~0.01% of BM-MNCs), quiescent, and homogeneous population, phenotypically characterized as being CD34^+^/CD133^+^/CXCR4^+^/Lin^−^/CD45^−^, and express embryonic stem cell-specific markers, such as SSEA-4 and TRA-1-81 on their surface and Oct-4, NANOG, and Sox2 transcription factors at the protein level [126]. Under steady state, VSELs are highly quiescent because they express low levels of genes involved in proliferation and cell signalling; that however become upregulated during cell activation. They have also been identified in cord blood [127]. They circulate in very small numbers in peripheral blood under steady state throughout life [128], but can be mobilized in larger numbers by G-CSF from the BM into the PB [129] and expanded ex-vivo [130]. VSELs are also physio-pathologically mobilized into PB in response to tissue injury following AMI [131], stroke [132], or critical leg ischemia [133]. VSEL-derived cells show vasculogenic potential as they trigger post-ischemic revascularisation in immune-deficient mice and acquire an endothelial phenotype either in vitro or in vivo [134]. Other in vivo experimental models have shown that injected purified VSELs contribute to hematopoiesis, angiogenesis, osteogenesis, as well as to myocardium and liver [135]. Thus CD34^+^ VSELs isolated from adult tissues appear to be “true” pluripotent stem cells, which could be used, through their progeny, to regenerate damaged organs (Fig. 1), and which may solve the problems inherent in the use of controversial embryonic stem cells or induced pluripotent stem cells (iPSCs). As a side effect, one can also send back to back Orlic [2] and his main detractors [62, 63]: if HSCs – that only represent one of the various CD34^+^ cell sub-populations - cannot effectively transdifferentiate into cardiac or endothelial progenitor cells, other CD34^+^ VSELs progenies can do it.

However, cardiac-repair mechanisms of CD34^+^ VSEL-derived subpopulations are still unclear. The largest contribution to neo-vascularization and repopulation of the ischemic scar likely comes from endothelial and cardiomyocytic CD34^+^ progenitor cells, as we and others have previously reported [20, 21, 25, 28, 30]. Additionally, injected CD34^+^ stem cells may release soluble paracrine factors and exosomes that can respectively enhance the proliferation of resident cardiomyocytes [135] or neo-angiogenesis [136]. CD34^+^ stem-cell commitment to the endothelial and cardiac pathways is strongly dependent on changes that occur in myocardial stiffness after AMI [137]. Cell commitment also likely depends on the release of a complex blend of cardioactive cytokines by inflammatory cells from the scar, reducing fibrosis, and avoiding remodelling effects [138–140]. In other words, such commitment does not occur under steady-state conditions, and committed cells cannot sufficiently benefit from these physical micro-environmental changes, as they are already lineage-specified and lack long-term self-renewal capacity. Wang et al. used an experimental nude-mouse model to show that human CD34^+^ cells persist in the injured heart for up to one year after reinjection and contribute to functional recovery [31]. A cell-to-cell communication of CD34+ cells with cardiac myocytes by nanotubes might also contribute to their acquisition of a cardiomyogenic phenotype [141]. In addition, the hypoxic environment of the infarct zone increases vascular endothelial growth factor (VGEF) expression by transplanted cells, which may accelerate the proliferation of endothelial cells and α-SM actin-positive cells (reviewed in [142]).

### Doses of Cells to be Delivered

Large numbers of cells are necessary, as demonstrated in many clinical reports concerning the use of BM-MNCs containing an insufficient number of CD34^+^ cells, even when up to 600 × 10^6^ MNCs were re-injected. Even if high CD34^+^ stem cell doses are delivered, as in our pilot study, heart function progressively improves over months, corresponding to the time necessary for stem cells to repopulate the infarcted area with their progeny through successive stages of cell division and maturation [25]. Several dose-ranging studies have showed that a significant improvement of heart function occurred from a threshold dose ≥10 × 10^6^ CD34^+^ cells in AMI [24, 35, 37]. In our opinion, the more stem cells delivered, the better will be the clinical results, at least in AMI, given beating heart–related mechanical loss, cell escape, and in situ cell apoptosis and death [85–87]. The situation is less clear for CHF and RA, as conflicting results have been reported [38, 143].

The best way to harvest a large number of CD34^+^ cells has been, up to now, to perform LKP after G-CSF mobilization. However, we have developed a proprietary expansion process that allows GMP production of up to 150 × 10^6^ CD34^+^ stem cells capable of both long-term self-renewal and lineage differentiation, starting from a simple autologous blood draw [144].

### Delivery Route

According to experimental studies, cells injected directly into the damaged myocardium are probably retained more efficiently than cells infused via the coronary arteries, which are limited by their weak homing capacity to the injured area [75]. Not only does this procedure appear to be as feasible and safe as IC injection, but also several meta-analyses confirm a more significant clinical improvement of post-AMI heart function after trans-endocardial than IC cell delivery, several of them even concluding that the latter had no effect on clinical events or changes in LV function or remodelling [89, 145]. As the reduction of scar size and ventricular functional responses preferentially occur at the sites of IM injection versus non-injected sites [146], performing 10 to 15 injections around or into (when technically feasible) the scar likely allows better intra-tissue diffusion. The use of a catheter that can be fixed into the cardiac tissue before each cell injection, such as the Helix™ (Biocardia) or C-Cathez™ (Celyad, Mont Saint-Guibert, Belgium) catheters would also enhance cell retention. Increasing the resistance of transplanted cells to ischemia or apoptosis, currently being tested in various experimental pre-conditioning models, might also improve their survival and subsequent functional recovery [147]. It is thus conceivable that better IM targeting of the cells could contribute to a true clinical benefit, making this route of delivery one of the key factors in cellular cardiac therapy.

### Optimal Time for Injection

Various reports and meta-analyses strongly suggest that the optimal time for cell delivery is likely between the eight day and the end of the second month after AMI. Aside from the physio-pathological reasons that justify this timeframe, it might also be dangerous to perform direct cell injection into the ischemic area before the end of the second week, as the injured myocardium is too weak within the immediate post-AMI period to be safely injected.

### Stage of Heart Disease

Currently available treatments do not help patients with chronic IHF or RA and there is a serious lack of emerging conventional therapies. Thus, IHF and RA represent a potential target for cell therapy. Although most benefits of cell therapy have been mainly observed in AMI patients with the most severely depressed LVEF at baseline, this does not appear to be the case in CHF. Globally, improvements in heart function in this indication have been extremely modest, if any, regardless of the clinical study or the cell type delivered. Clearly, it is difficult to imagine how stem cells injected directly into a fibrotic/calcified scar could survive. Moreover, at this stage, the scar no longer releases cardioactive cytokines that likely facilitate in situ retention, multiplication, and differentiation of the injected cells [138–140]. However, when cells are injected in the scar border, they may have a neo-angiogenic effect on the hibernating myocardium surrounding the scar, thus attenuating its remodelling. MSCs might be more efficient in this context [53, 146]. As IHF is characterized by progressive volume dilatation and cardiac myasthenia, it might actually be preferable to inject very large amounts of stem cells (preferentially MSCs?) via the common coronary artery trunk to allow their spread into all areas of the heart.

## General Conclusion

**Fig. 2:**
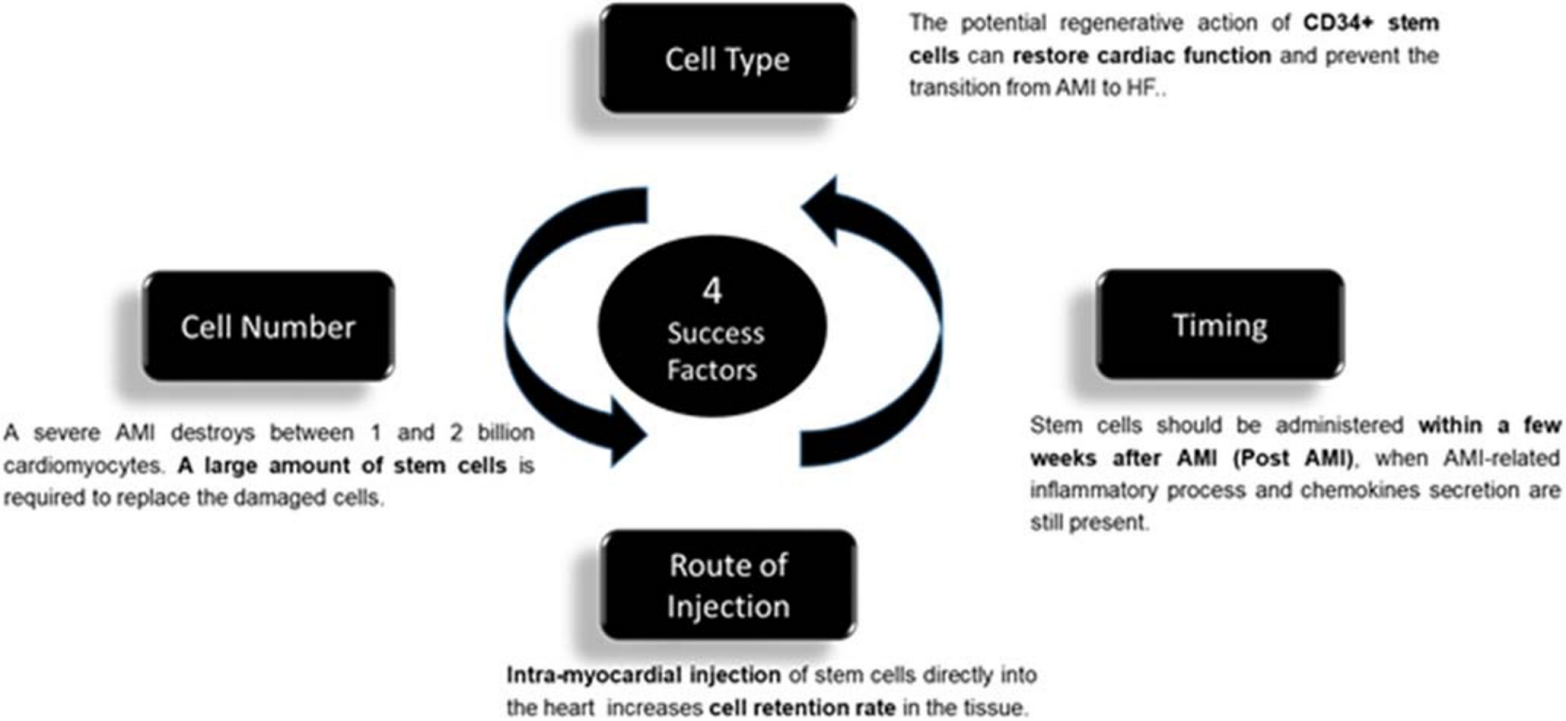
Key factors that prevent secondary heart failure. The four important points are CD34^+^ stem cells; high cell doses; intramyocardial injection route; and acute or sub-acute myocardial ischemia indication

Successful cell therapy in acute ischemic heart diseases likely depends on direct injection into or at the border of the ischemic lesion of the largest number possible of CD34^+^stem cells via an appropriate catheter, between 15 days and two months after severe AMI. These four points: CD34^+^ stem cells; high cell doses; intramyocardial injection route; and acute or sub-acute myocardial ischemia indication, appear as to be the key factors that allow avoiding secondary heart failure, which is very difficult to effectively treat and is associated with a short/middle term bad prognosis (Fig. 2). Until now, none of the reported clinical trials have associated all of these key factors. This is the objective of the on-going EXCELLENT trial (EUDRACT 2014–001476-63) using autologous PB-CD34^+^ cells, expanded via the automated StemXpand® device and StemPack® production kits we have developed, and reinjected trans-endocardially via the Helix™ catheter at the end of the fourth week post-AMI.
